# Interobserver variability in organ at risk delineation in head and neck cancer

**DOI:** 10.1186/s13014-020-01677-2

**Published:** 2021-06-28

**Authors:** J. van der Veen, A. Gulyban, S. Willems, F. Maes, S. Nuyts

**Affiliations:** 1 Department of Oncology, Radiation-Oncology, University of Leuven, University Hospitals Leuven, 3000 Leuven, KU Belgium; 2 Department of Medical Physics, Jules Bordet Institute, Brussels, Belgium; 3 Department ESAT, Processing Speech and Images (PSI), Medical Imaging Research Center, KU Leuven, University Hospitals Leuven, 3000 Leuven, Belgium

**Keywords:** Head and neck, Interobserver variability, Contouring, Organs at risk, Guidelines

## Abstract

**Background:**

In radiotherapy inaccuracy in organ at risk (OAR) delineation can impact treatment plan optimisation and treatment plan evaluation. Brouwer et al. showed significant interobserver variability (IOV) in OAR delineation in head and neck cancer (HNC) and published international consensus guidelines (ICG) for OAR delineation in 2015. The aim of our study was to evaluate IOV in the presence of these guidelines.

**Methods:**

HNC radiation oncologists (RO) from each Belgian radiotherapy centre were invited to complete a survey and submit contours for 5 HNC cases. Reference contours (OARref) were obtained by a clinically validated artificial intelligence-tool trained using ICG. Dice similarity coefficients (DSC), mean surface distance (MSD) and 95% Hausdorff distances (HD95) were used for comparison.

**Results:**

Fourteen of twenty-two RO (64%) completed the survey and submitted delineations. Thirteen (93%) confirmed the use of delineation guidelines, of which six (43%) used the ICG. The OARs whose delineations agreed best with the OARref were mandible [median DSC 0.9, range (0.8–0.9); median MSD 1.1 mm, range (0.8–8.3), median HD95 3.4 mm, range (1.5–38.7)], brainstem [median DSC 0.9 (0.6–0.9); median MSD 1.5 mm (1.1–4.0), median HD95 4.0 mm (2.3–15.0)], submandibular glands [median DSC 0.8 (0.5–0.9); median MSD 1.2 mm (0.9–2.5), median HD95 3.1 mm (1.8–12.2)] and parotids [median DSC 0.9 (0.6–0.9); median MSD 1.9 mm (1.2–4.2), median HD95 5.1 mm (3.1–19.2)]. Oral cavity, cochleas, PCMs, supraglottic larynx and glottic area showed more variation. RO who used the consensus guidelines showed significantly less IOV (p = 0.008).

**Conclusions:**

Although ICG for delineation of OARs in HNC exist, they are only implemented by about half of RO participating in this study, which partly explains the delineation variability. However, this study highlights that guidelines alone do not suffice to eliminate IOV and that more effort needs to be done to accomplish further treatment standardisation, for example with artificial intelligence.

**Supplementary information:**

**Supplementary information** accompanies this paper at 10.1186/s13014-020-01677-2.

## Purpose

Radiotherapy (RT) is an important treatment modality in the fight against head and neck cancer (HNC) where efforts are continuously being made to improve disease outcome without increasing toxicity. Intensification of RT [1] and/or concomitant chemotherapy [2], have improved survival, however with more acute and late toxicity [3]. Unfortunately, loco-regional failure rates remain high with approximately 30% loco-regional recurrences over 5 years, which impacts morbidity and mortality [4, 5]. The ultimate aim is to deliver an as high as possible dose to the target volumes (TVs) to achieve disease control whilst keeping the dose to normal surrounding tissue as low as possible, to limit toxicity. The complex anatomy of the head and neck however makes this very challenging because of the close proximity between TVs and organs at risk (OARs) [6]. A huge step forward in realising this was the implementation of more conformal techniques such as intensity modulated radiotherapy (IMRT) and volumetric arc therapy (VMAT) which allow better sparing of OARs resulting in a decrease in toxicity and a better quality-of-life [7–10]. To fully utilise these benefits, accurate and consistent delineation of TVs and OARs is crucial as it determines where the high dose should be delivered and it is necessary to produce an optimal, patient specific dose plan. Inaccuracies in this step can have a detrimental effect on treatment outcome either by unnecessarily giving a too high dose to normal tissue which could result in more toxicity, or by inadequately treating the TVs which could result in loco-regional treatment failure [11]. Delineation accuracy is significantly limited by interobserver variability (IOV) in delineation of TVs [11–16] and OARs [11, 17] and should be minimised to improve treatment standardisation to provide the best quality of care possible for patients. Furthermore, IOV has an impact on the interpretation of radiation induced toxicity and could therefore also have an impact on the outcome of multicentre trials (11). International consensus guidelines (ICG) describing the delineation of 25 OARs in the head and neck were published in 2015 by Brouwer et al. [18] after IOV had been shown between 5 radiation oncologists (RO) [17].

An initiative was launched to map the RT landscape within Belgium for HNC, regarding delineation of TVs [16] and OARs, in the presence of ICG [18]. Since the publication of these ICG, this is the first study of its kind to identify (a) which guidelines are used, (b) which OARs are delineated in clinical practice and (c) the extent of IOV in organ at risk (OAR) delineation, with the cooperation of multiple RO from different RT centres.

## Methods and materials

### Study design

In February 2017, all 25 RT centres in Belgium were invited to participate in this study. One experienced HNC RO from each participating centre was asked through an online survey which guidelines they used for delineation of OARs and whether these guidelines in their opinion needed a revision or clarification (survey in Additional file 1: survey questions and answers). The same RO was also invited to submit OAR delineations of five previously selected HNC cases (Additional file 2: Table 1 Patient characteristics). These cases were selected to represent different tumour sites and different tumour and nodal stages, excluding post-operative patients and patients with scatter artefacts on planning CT. We refer to our previous study for a full description of each case [16], which was also provided to each participating RO, including detailed information on clinical examination, diagnostic imaging (MRI, CT, PET-CT) and biopsy.

**Table 1:** Median and range of DSC, MSD and HD95 per OAR

	DSC			MSD (mm)			HD95 (mm)			Nr. of contours by RO using Brouwer et al. guidelines (max = 27)	Nr. of contours by RO using no/other guidelines (max = 35)
	Median	Range		Median	Range		Median	Range			
		Min	Max		Min	Max		Min	Max		
Brainstem	0.88	0.61	0.92	1.5	1.1	4.0	4.0	2.3	15.0	24 (89%)	31 (89%)
Cochlea left	0.45	0.00	0.79	1.9	0.8	11.0	3.7	1.9	14.9	16 (59%)	9 (26%)
Cochlea right	0.38	0.00	0.83	2.1	0.9	10.5	4.1	1.5	14.4	16 (59%)	9 (26%)
Glottic area	0.45	0.17	0.87	2.8	0.9	8.4	9.4	1.8	18.5	13 (48%)	10 (29%)
Mandible	0.90	0.79	0.94	1.1	0.8	8.3	3.4	1.5	38.7	26 (96%)	29 (83%)
Oral cavity	0.77	0.45	0.91	4.6	1.8	11.6	14.5	4.3	30.1	19 (70%)	23 (66%)
Parotid left	0.82	0.62	0.88	1.9	1.2	4.2	4.9	3.1	16.5	27 (100%)	35 (100%)
Parotid right	0.83	0.51	0.90	2.0	1.4	4.9	5.1	3.2	19.2	26 (96%)	35 (100%)
PCM-inferior	0.53	0.00	0.78	2.9	1.1	16.8	12.2	2.6	78.0	12 (44%)	6 (17%)
Middle	0.53	0.29	0.82	3.8	0.9	13.4	12.1	2.0	36.0	12 (44%)	7 (20%)
Superior	0.50	0.19	0.73	2.6	1.3	6.7	10.5	3.2	27.5	12 (44%)	7 (20%)
Spinal cord	0.78	0.56	0.90	2.2	0.8	10.4	12.1	1.7	72.1	17 (63%)	34 (97%)
SMG left	0.87	0.57	0.91	1.3	0.9	3.4	3.1	1.8	12.2	26 (96%)	24 (69%)
SMG right	0.87	0.72	0.92	1.2	0.9	2.5	3.1	1.9	10.1	27 (100%)	24 (69%)
Supragl. larynx	0.65	0.11	0.86	3.5	1.0	13.3	9.6	3.0	28.7	11 (41%)	9 (26%)

Median and range were calculated over all delineations made by all radiation oncologists on all cases. The number of delineations varies for each OAR as shown in the 2 right columns. Radiation oncologists who use the guidelines from Brouwer et al. delineate the cochleas, glottic area, PCSM,s, SMG and supraglottic larynx more than other radiation oncologists. The spinal cord however was delineated less, because 2 RO delineated the spinal canal instead

*DSC* Dice Similarity Coefficient, *HD95* 95% Hausdorff distance, *mm* millimetre, *MSD* mean surface distance, *PCM* pharyngeal constrictor muscles, *RO* radiation oncologist, *SMG* submandibular gland, *Supragl* supraglottic

A planning CT scan was acquired in supine position after iodine containing contrast medium (Visipaque 320®) was injected intravenously. For further details regarding the planning CT, we refer to our previous publication [16]. The anonymized planning CTs with delineated gross tumour volume of the primary tumour (GTVp) and pathological lymph nodes (GTVn) were provided and dedicated software (Aquilab Software, Lille, France) was used for secure data transfer to and from each participating centre.

A reference contour of each OAR (OARref) was created for comparison, with the help of an in-house developed auto-delineation tool to ensure consistent delineations [19]. This tool was created using deep learning based on a training set of HNC planning CTs delineated according to the ICG [18]. The tool has been validated and implemented in our clinical practice [20] and has been shown to decrease IOV in our centre. The auto-delineation contours were carefully reviewed and manually corrected if needed to remove minor mistakes.

### Delineation agreement analysis

Pair-wise agreement of the 3D set of contours submitted by each RO to the corresponding reference contours made according to the ICG (OARref) was assessed for each OAR separately using Dice similarity coefficient (DSC), mean surface distance (MSD) and the 95% Hausdorff Distance (HD95). The DSC was calculated as the ratio of the volume of overlap of both contour sets (A and B), divided by their total volume:

$$DSC=2*\frac{\left|A\cap B\right|}{\left|A\right|+\left|B\right|}$$

A perfect overlap between contours results in DSC = 1, while no overlap results in DSC = 0. Clinical interpretation of intermediate DSC values is complicated by the fact that DSC is biased with regards to volume (i.e. structures with larger volume yielding higher DSC than smaller structures with similar absolute volume difference) [21]. Hence, also MSD and HD were calculated which are distance measures. MSD is the mean distance between the surface of the contours of the RO and the OARref. HD is the maximum of the 3D distances between any two closest points on each of both OAR contours, which is independent of their volume. Instead of the maximum distance which is sensitive to outliers, we report HD95, i.e. the 95th percentile. For MSD and HD95, a smaller value corresponds to more delineation agreement compared to a larger value. Median DSC, MSD and HD95 were computed for each OAR separately to asses difference in IOV per OAR. To assess the impact of the guidelines the RO used on IOV, DSC, MSD and HD95 were computed separately for the two groups. An independent, two-sided T-test was used to quantify significance, *P* < 0.05 was considered statistically significant.

## Results

Three RO encountered technical problems and could therefore not take part in this study. Fourteen of the remaining 22 RO (64%) responded to the questionnaire and submitted at least one delineation. Eleven RO delineated all 5 patients, 1 delineated 3 cases and 2 delineated 2 cases (62 cases in total). Of the fourteen RO, four worked in a university hospital and ten in a general hospital. Three hospitals were public hospitals, the remaining eleven were private,

### Survey

Thirteen of fourteen participating RO confirmed using guidelines for OAR delineation of which six used the ICG of Brouwer et al. [18] and one also used the publication of Christianen et al. [22]. One RO used the publication of Genovesi et al. [23], while six did not specify which guidelines they used. Seven RO found an update or clarification of existing guidelines, or creation of new guidelines necessary. Five of these did not use the ICG and two did (Additional file 1).

### DSC, MSD, HD95 and volumes

**Fig. 1:**
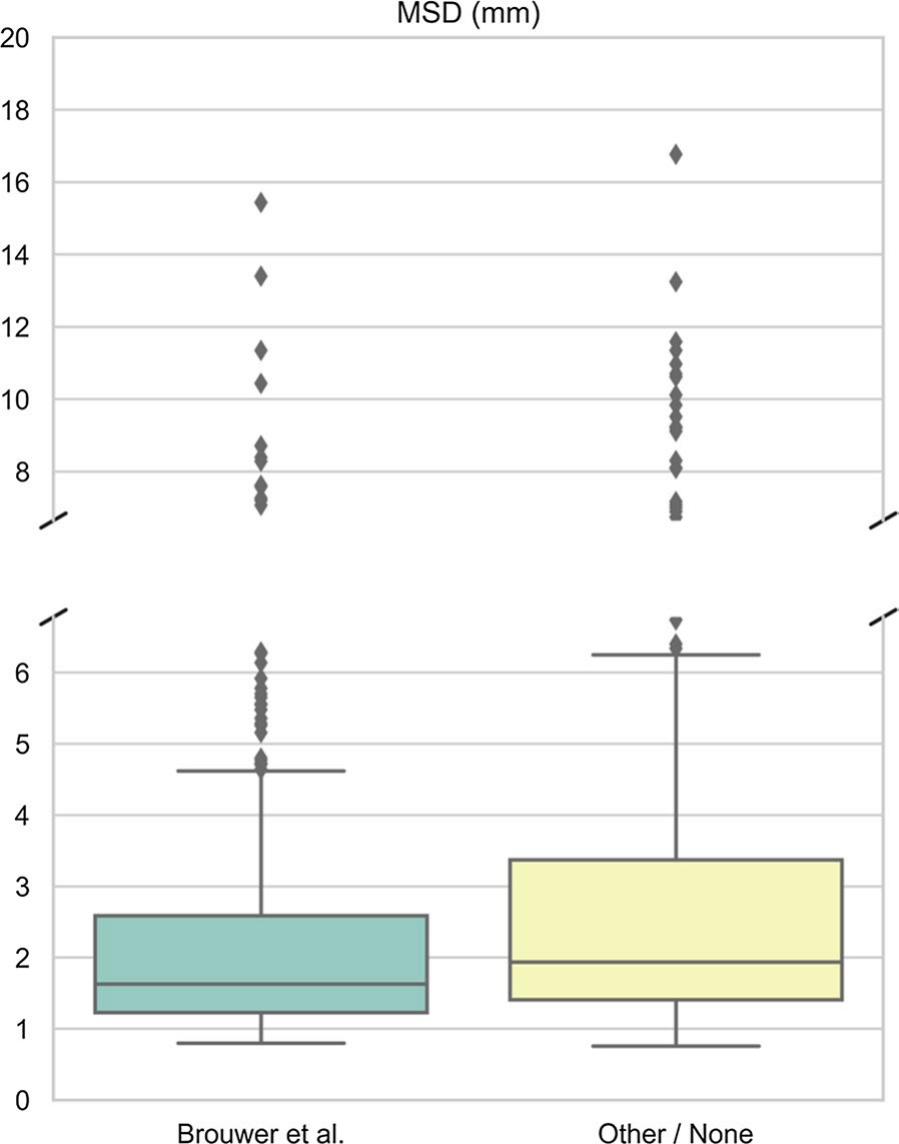
Overall mean surface distance. The boxplot shows better agreement with the reference contour when the ICG from Brouwer et al. are used by the RO compared to other RO (p = 0.008). *mm* millimetre, *ICG* international consensus guidelines, *MSD* mean surface distance, *RO* radiation oncologists

**Fig. 2:**
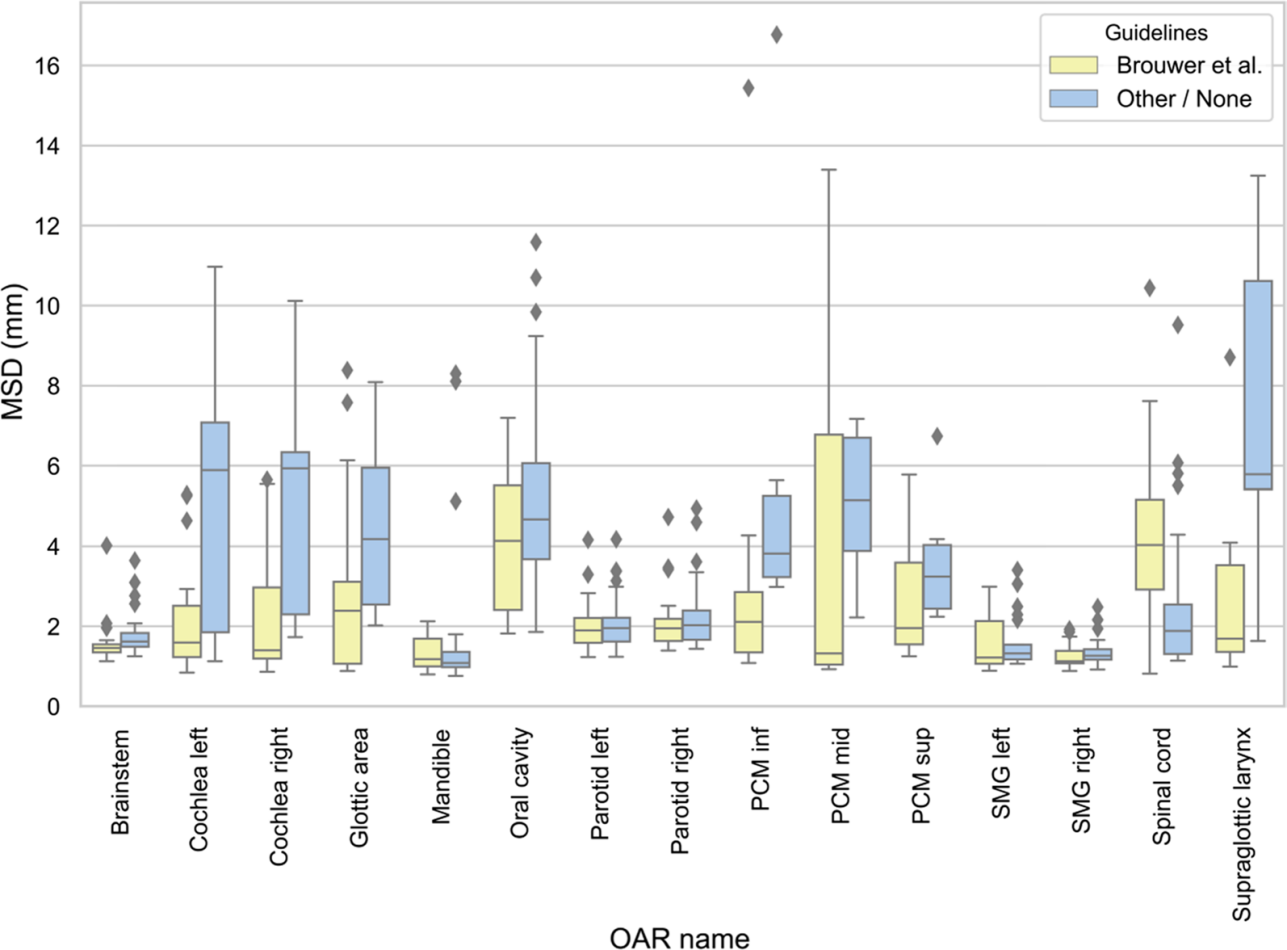
Mean surface distances for the different OARs. This figure shows better agreement with the reference contour when the guidelines from Brouwer et al. are used (yellow). Diamond shape markers represent outliers (more than 1.5 × interquartile range above the upper quartile and below the lower quartile). *mm* millimetre, *MSD* mean surface distance, *PCM* pharyngeal constrictor muscle, *SMG* submandibular gland

Table 1 shows the median DSC, MSD and HD95 per OAR and range for all OARs, for all 5 patients. Median DSC ranges from 0.38 (left cochlea) to 0.90 (mandible), median MSD ranges from 1.1 mm (mandible) to 4.6 mm (oral cavity) and median HD95 from 3.1 mm (submandibular glands) (SMGs) to 14.5 mm (oral cavity). Figure 1 shows the overall difference in MSD between RO who use the ICG versus other RO and Fig. 2 shows the differences per OAR. They show that MSD is significantly smaller when the ICG are applied (p = 0.008). In Additional file 3: Fig. 1, DSC and corresponding MSD for each OAR are shown separately to show that some OARs show more IOV than others. Additional file 4: Fig. 2 shows the difference between the two RO groups for DSC and HD95. Additional file 5: Fig. 3 shows the range of volumes delineated per patient and per OAR compared to OARref.

**Fig. 3:**
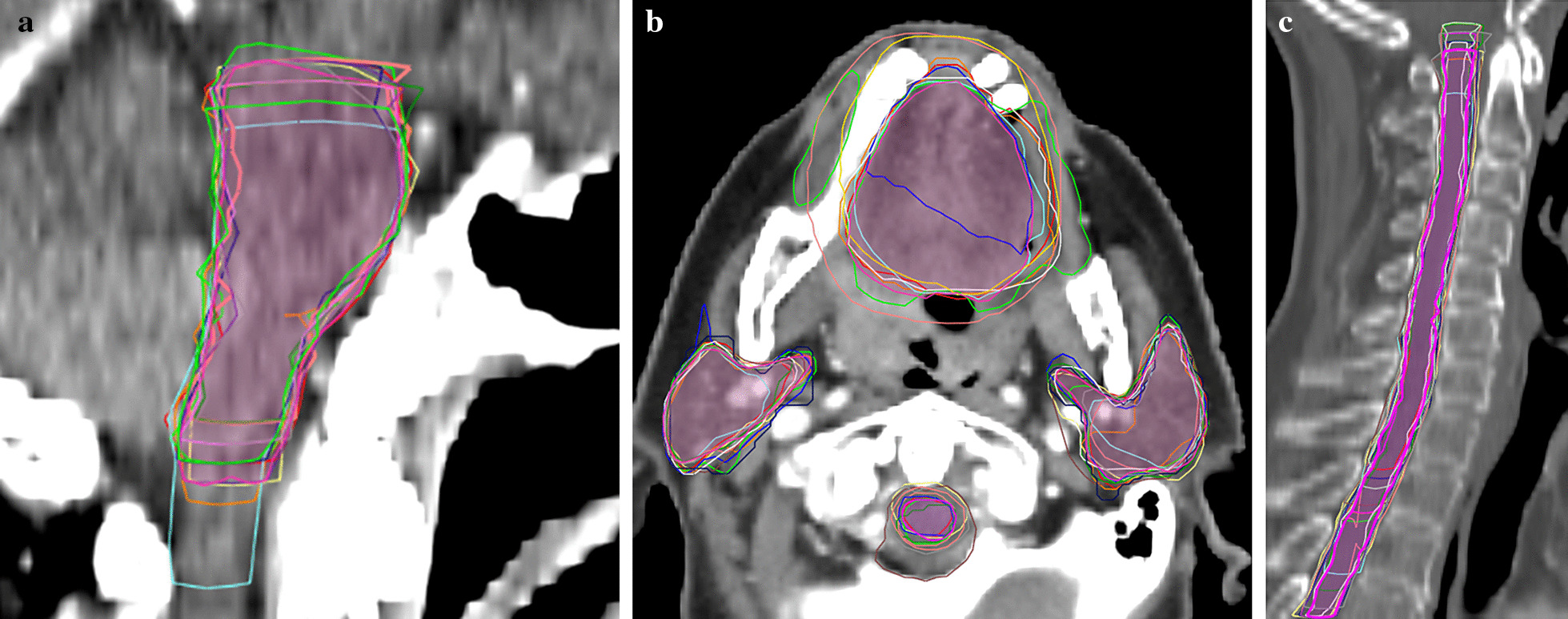
CT images showing different OAR contours Reference contours according to the ICG (lilac) vs delineations from the different RO. **a** brainstem (sagittal plane): difference in cranial and caudal borders; **b** oral cavity, spinal cord and PG (axial plane): Inclusion of buccal mucosa (green contour) and teeth (orange and pink contours) by some RO. Variation in spinal cord and PG contours; **c** spinal cord (sagittal plane): difference in cranial and caudal borders. *ICG* international consensus guidelines, *PG* parotid glands, *RO* radiation oncologist

### Brainstem

The brainstem was delineated in 89% of cases (no difference between the two RO groups). Most RO in this study started delineation in the most cranial slice where the brainstem was visible. The caudal border differed with a few slices between RO but was mostly according to the guidelines (Table 2, Fig. 3a). The circumferential contour on the axial plane showed little variation (Additional file 6: Fig. 4a). On visual inspection of the contours, there was no clear difference between the two groups of RO.

**Table 2:** Short description of delineation guidelines per organ at risk, the errors delineated and possible consequences

Organ at risk	International consensus guidelines [18]	Errors	Consequences of errors
Brainstem	Cranial: bottom section of the lateral ventricles Caudal: tip of the dens of C2	Cranial: start when the brainstem becomes visible (all RO) Caudal: different interpretation of “tip” so a few slices difference	No impact on relevant dose parameter for precise NTCP (Dmax), depending on tumour location. But can effect estimated dose to spinal cord which has a more strict dose constraint
Cochlea	CT scan in bone setting, cochlea in temporal bone	Incorrect location (2 RO) Entire petrous part of the temporal bone delineated (3 RO)	Effect on dose when wrong location is delineated Difference in volume can have an impact on Dmean and Dmax depending on tumour location
Glottic area	Cranial: cranial tip of arytenoids Caudal: caudal edge of thyroid cartilage Posterior: cricoid, anterior border of arytenoids Enclosed by the thyroid cartilage	Entire larynx delineated, starting caudal of the hyoid and including the thyroid cartilage and arytenoids (2 RO) Included caudal part of supraglottic larynx (2 RO) Included arytenoids (2 RO)	Impact on relevant dose parameter for precise NTCP (Dmean)
Mandible	CT scan in bone setting Excluding teeth, including mandibular condyles and coronoid process	Teeth included (not systematically, but some RO in some cases) Mandibular condyles and coronoid process not included (1 RO systematically)	No impact on relevant dose parameter for precise NTCP (Dmax), depending on tumour location
Oral cavity	Cranial: Hard palate mucosa and mucosal reflections near the maxilla Caudal: base of tongue mucosa and hyoid posteriorly and the mylohyoid and anterior belly of the digastric muscle anteriorly Anterior and lateral: inner side of the mandible and maxilla Posterior: soft palate, uvula and base of tongue	Included teeth (two RO) Included the buccal mucosa (1 RO) Excluded posterior part of oral cavity (1 RO) Excluded base of tongue (1 RO)	Impact on relevant dose parameter for precise NTCP (Dmean)
Parotid gland	Cranial: external auditory canal, mastoid process Caudal: posterior part submandibular space Anterior: Masseter muscle, posterior border mandibular bone, pterygoid muscle (med. and lat.) Posterior: sternocleidomastoid muscle, lateral side posterior belly digastric muscle Lateral: platysma and subcutaneous fat Medial: Posterior belly of the digastric muscle, styloid process, para-pharyngeal space	Cranial and caudal borders varied up to a few slices between RO Anterior: sometimes inclusion of the masseter and pterygoid muscles Medial: digastric muscle sometimes included	Impact on relevant dose parameter for precise NTCP (Dmean)
PCM inferior [21]	Caudal: lower edge of arytenoids Cranial: first slice caudal to the hyoid bone	Caudal: several slices difference between RO	Impact on relevant dose parameter for precise NTCP (Dmean)
PCM middle [21]	Cranial: cranial border of C3 Caudal: caudal border of hyoid bone	Cranial: cranial border stopped at caudal border of C3 (3 RO). Only 2 RO delineated cranially enough	Impact on relevant dose parameter for precise NTCP (Dmean)
PCM superior [21]	Cranial: tips of the pterygoid plates (hamulus) Caudal: caudal border of C2	Cranial: 1 RO delineated the superior muscle up to the base of skull	Impact on relevant dose parameter for precise NTCP (Dmean)
Spinal cord	Spinal cord, not canal Cranial: tip of dens of C2 Caudal: At least cranial edge of T3, lower for more caudal tumours	Spinal canal delineated (2 RO) Cranial: some difference depending on what was interpreted as “tip” of dens of C2 Caudal: not caudal enough, stopped several slices too high (3 RO, each in one patient)	No impact on relevant dose parameter for precise NTCP (Dmax), depending on tumour location. But can effect estimated dose to brainstem which has a less strict dose constraint
Submandibular gland	Cranial: medial pterygoid muscle and mylohyoid muscle Caudal: fatty tissue Anterior: lateral surface mylohyoid muscle and hyoglossus muscle Posterior: para-pharyngeal space, sternocleidomastoid muscle Lateral: medial surface medial pterygoid muscle, mandible, platysma Medial: lateral surface mylohyoid muscle, hyoglossus muscle, superior and middle PCM, anterior belly digastric muscle	Cranial: some variation in cranial border between RO	Impact on relevant dose parameter for precise NTCP (Dmean)
Supraglottic larynx	Cranial: tip of epiglottis Caudal: cranial edge of arytenoids Posterior: inferior PCM, pharyngeal lumen Confined by the thyroid cartilage and hyoid bone, exclude pharyngeal lumen	Cranial: entire pharyngeal lumen delineated at the tip of the epiglottis (1 RO) Caudal: delineation 2–3 cm more caudal than guidelines (2 RO)	Impact on relevant dose parameter for precise NTCP (Dmean)

For detailed description of contour guidelines, see Brouwer et al. [18] and Christianen et al. [21]

*NTCP* normal tissue complication probability, *Dmean* mean dose, *Dmax* maximum dose, *PCM* pharyngeal constrictor muscle, *RO* radiation oncologist

### Cochlea

Cochleas were delineated in 40% of cases (59% with ICG vs 26% without). Disagreement of contours was small, although 3 RO delineated the entire petrous part of the temporal bone, one of whom used the ICG (Additional file 6: Fig. 4b) and 2 who did not use the ICG delineated a region that did not contain the cochlea in one patient each (Additional file 6: Fig. 4c).

### Glottic area

It was delineated in 48% of cases by RO who used the ICG compared to 29% of RO who did not. It was delineated more in patients with oropharyngeal tumours (58%) than in patients with laryngeal, supraglottic or hypopharyngeal tumours (22%). Two RO delineated the entire larynx starting caudal of the hyoid bone and included the thyroid cartilage and arytenoids. One RO included part of the supraglottic larynx, another included the arytenoids and a third included both. Three RO delineated the glottic area according to the ICG, and all three confirmed using the guidelines in the survey (Additional file 6: Fig. 4d+e).

### Mandible

Vast majority (89%) of the submissions included a delineation of the mandible (96% with ICG vs 83% without). There were minor differences on visual inspection compared to OARref although sometimes the teeth were included as well (Additional file 6: Fig. 4f). One RO did not include the mandibular condyles and coronoid process.

### Oral cavity

Two thirds (68%) of the submissions included the oral cavity (70% with ICG vs 66% without). Two RO included the teeth (one used the ICG), and one RO who used the ICG included the buccal mucosa (Fig. 3b). The cranial border was consistently selected as the mucosa of the hard palate, but the posterior and caudal border showed more variation (Additional file 6: Fig. 4g). One RO excluded the posterior part of the tongue, and another the base of tongue.

### Parotid glands

The parotid glands (PGs) were delineated most often by all RO. Only one right parotid gland was not delineated by one RO for an unknown reason. At the anterior border the masseter and pterygoid muscles were sometimes included and at the medial border the digastric muscle (Fig. 3b + Additional file 6: Fig. 4h). The cranial and caudal borders varied up to a few slices.

### Pharyngeal constrictor muscles

The three pharyngeal constrictor muscles (PCMsup, PCMmid, PCMinf) were delineated by 9 RO, but only by 5 separately. RO who used the ICG delineated the PCMs more often than other RO, 44% vs. 20%. There was good agreement in the cranial border of PCMsup, although one RO delineated it up to the base of skull. It also showed variation in the anterior border (Additional file 6: Fig. 4i). Regarding PCMmid, only two RO delineated cranially enough, the others stopped at caudal level C3 (Additional file 6: Fig. 4j). There was good consensus regarding the cranial border of the PCMinf but the caudal border differed with multiple slices between RO. There was good agreement in the lateral extension of the contours in all three muscles.

### Spinal cord

The spinal cord was delineated in 82% of cases (62% with ICG vs 97% without) and the spinal canal in the other cases (two RO who both used the ICG and once by a RO in the other group) (Fig. 3b). Besides this, the largest differences were seen in the cranial border (depending on the caudal border of the brainstem) and the caudal border (Fig. 3c). Some RO delineated the spinal cord all the way to the most caudal slice of the CT scan, others stopped several slices higher. Three RO stopped a few slices cranial to T3 in one patient each.

### Submandibular glands

The SMGs were delineated in 81% of cases (98% with ICG vs 69% without ICG). Good agreement was seen between all RO (Additional file 6: Fig. 4k), except in the cranial border (Additional file 6: Fig. 4l).

### Supraglottic larynx

The supraglottic larynx was delineated by less than half of the RO in patients with an oropharyngeal tumour, and by less than a quarter of RO in patients with a laryngeal, supraglottic or hypopharyngeal tumour. In total it was delineated at least once by seven RO and more often when the ICG were used (41% vs 26%). Two RO systematically delineated 2–3 cm more caudally then the guidelines suggest (Additional file 6: Fig. 4m) and one RO more cranially (Additional file 6: Fig. 4n).

## Discussion

The present study shows that even though there are ICG for OAR delineation, these are not consistently applied by all HNC RO in routine clinical practice. This results in variability in terms of which OARs are delineated and how these are delineated. Furthermore, we have shown that even when they are implemented, there is still room for improvement regarding IOV. This is in line with what RO in this study indicate, namely half of them found that new or updated guidelines are necessary.

Previous studies have also shown significant IOV in delineation of several OARs such as the spinal cord, brainstem, PGs, glottic larynx and thyroid cartilage [11, 17, 24]. Consequently, ICG for OAR delineation were published in 2015 to try to standardise delineation of OARs [18]. The current study is the first one to investigate IOV between RO of different centres for a large set of OARs, since these ICG were published. We had similar results to Brouwer et al. [17], although DSC (or concordance index) was higher in our study which could imply improvement of IOV with the ICG as 6 of 14 RO used them. In a study on the benefits of deep learning for OAR delineation [20], we also showed IOV in OAR delineation between two RO from the same centre who both used the ICG. The IOV however was smaller than in the current study, and improved even more with the use of the automated delineation tool.

There are several reasons that could explain the contour variation between RO and the reference contour in the present study. A reason that has already been mentioned, is that different guidelines are used, either because the ICG [18] were not known to exist, or because other guidelines were used. The effect of using the ICG could clearly be seen on several OARs, namely the cochleas, glottic area, PCMs and supraglottic larynx, which were delineated more often and with better agreement. Figures 1 and 2 support this hypothesis because MSD is significantly smaller for the RO using the ICG compared to the other group (p = 0.008). However, even when the ICG are used, there was still IOV compared to the reference contours. A first possible reason is that the edges of the OARs may be unclear/blurry on CT (PCMs, anterior and medial borders of PGs), needing interpretation by the delineating RO, which can result in IOV. Secondly, different CT windowing can also have an impact on OAR visualisation, resulting in different volumes. Thirdly, the guidelines might be misunderstood or misinterpreted. For example the supraglottic larynx which should start cranially at the tip of the epiglottis was delineated by one RO including the air surrounding the tip (Additional file 6: Fig. 4n). The inclusion of air has a large impact on the volume delineated, which is also often seen in case of the oral cavity. Another misinterpretation occurs at the cranial and caudal borders, which often differed a few slices. For example at the caudal border of the brainstem, because the “tip of the dens of C2” can be prone to misinterpretation (Fig. 3a). Also the spinal cord showed variation in the caudal border because some RO delineated it all the way to the most caudal slice of the CT, and others stopped more cranially. Two RO who used the ICG delineated the spinal canal instead of the spinal cord so these were excluded from the analysis which resulted in less delineations (Table 1) and less agreement (Fig. 2). Not only the delineated volumes differed, but also whether the OAR was delineated or not varied significantly. The mandible, brainstem, spinal cord, salivary glands and oral cavity were consistently delineated in all patients, irrespective of which RO delineated them. But several OARs seem less well-known, especially to RO who did not use the ICG. This resulted in less than half of them to delineate the cochleas, glottic area, PCMs and supraglottic larynx. Even the RO using the ICG did not always delineate the OARs described in the guidelines, even though they did delineate them more often (Table 1). A reason for this could be that the RO may have deemed delineation of the OAR unnecessary for treatment planning because the tumour was situated far away or too close to spare the OAR anyway.

Nelms et al. [25] showed the impact of OAR contouring variation on dose volume histograms (DVH) and concluded that differences in maximum dose (Dmax) and mean dose (Dmean) per OAR could be large, depending on the degree of IOV and the RT plan. On the one hand there are OARs where Dmax can be used for plan optimisation (mandible, brainstem, spinal cord and cochleas) and for these OARs, precision of the contour (especially in cranial and caudal direction) may be less important because volume does not affect Dmax significantly. Exceptions of course are sub-optimal delineations, for example when OARs (such as cochleas in 2 patients in this study) are delineated in the wrong position. Additionally, the caudal border of the spinal cord is important for caudally located tumours and the cranial border of the spinal cord should also be delineated carefully, as the spinal cord has a stricter dose constraint than the brainstem. Shifting the border between these two OARs more caudally means the spinal cord could receive a higher dose than anticipated. On the other hand, there are OARs (salivary glands, oral cavity, PCMs, glottic area and supraglottic larynx) where Dmean is used for treatment planning and evaluation. In that case, the volume delineated is important because a smaller volume would result in a higher Dmean than a larger volume. Additional file 4: Fig. 2 shows that for the glottic area, oral cavity and supraglottic larynx, the smallest/largest volume contoured by RO is sometimes half/double the size of the OARref volume. A summary of the impact of sub-optimal delineations on dosimetry is listed in Table 2.

The consequences of inconsistent OAR delineation should not be underestimated as it is crucial for developing a treatment plan that represents reality. Incorrect or inaccurate delineation of OARs can impact DVH and could in turn impact normal-tissue complication probability (NTCP), affect evaluation of treatment plans and result in unexpected treatment-related morbidity. In turn, this could also affect the performance of predictive models and should be kept in mind in multicentre trials. Furthermore, care should be taken when using constraints from publications or other RO as these may have been developed with different OAR volumes, which could result in more unexpected toxicity. Correct delineation of OARs is also important to fully utilise the benefits of highly conformal techniques such as IMRT, VMAT and proton therapy, as incorrect delineation will counteract this benefit. Besides unexpected toxicity resulting from incorrect delineation of OARs, there is also the possibility of geographical misses. When delineating the clinical target volume, it may be adapted to exclude overlapping OARs which it does not invade. However, if the OAR is incorrectly delineated and the region is excluded from the clinical target volume or planning target volume, this could result in a geographical miss. Lastly, RO should be aware that even when identical guidelines are used, delineations still differ from one another (Fig. 1). We therefore advise regular joint delineation review sessions as a form of continuous training. If the guidelines would be updated, it would be useful to consider a general recommendation of mandatory and optional OARs to be delineated, in function of tumour location. In the future, it would also be useful if the preferred window level setting per OAR would be added to the guidelines, for optimal delineation. We also strongly believe there is a place for the automated delineation of OARs, as we have shown its benefits in reducing IOV and improving time efficiency in a previous study [20].

There are several limitations to the present study that should be addressed. Firstly, participation was voluntarily which could result in a response bias because not all invited clinical centres took part (64%). However, RO from university hospitals and general hospitals took part in the study. A second potential limitation is that not all RO answered which guidelines they used for delineation of OARs. Although this has no impact on the observed IOV, it does affect the perceived impact of the implementation of guidelines. Thirdly, participants were asked to delineate as they would do in clinical practice to give a realistic indication of therapeutic variability. This however meant that not all OARs were delineated by all RO, although it reflects variation in how patients are treated in reality. Lastly, reference contours were delineated using the ICG [18] and although this was done with the utmost care and with the help of an automated delineation tool, we cannot deny that this in itself required interpretation of the guidelines, which could introduce bias.

## Conclusions

Although ICG for delineation of OARs in HNC have been published several years ago, they are only implemented by half of RO participating in this study, which partly explains some of the delineation heterogeneity. Although there was less IOV between RO using the ICG, this study highlights that delineation guidelines alone do not suffice and that more effort needs to be done to accomplish further treatment standardisation, for example with the implementation of artificial intelligence tools for automated delineation.

## Supplementary information

**Additional file 1.** Survey and results from 14 radiation oncologists (RO).

**Additional file 2.** Patient characteristics.

**Additional file 3.** DSC and MSD of all OARS for all 5 patients. Every data point represents an organ at risk in one patient.

**Additional file 4.** Boxplots highlighting the differences between radiation oncologists using the guidelines from Brouwer et al., compared to radiation oncologists who use no or other guidelines. **a** Results of the dice similarity coefficient shows no significant difference between the two groups (p= 0.112). **b** Results for HD95 shows no significant difference between the two groups (p=0.219).

**Additional file 5.** The boxplots depict the variation in volumes delineated by the different radiation oncologists for each patient separately. The boxplot shows the interquartile range (IQR), the median (horizontal line) and the minimum and maximum volume delineated (whiskers). OARref shows the organ at risk volume delineated according to the international consensus guidelines of Brouwer et al.

**Additional file 6.** CT images showing interobserver variation for OAR contouring. The lilac volume is the reference delineation according to the guidelines (OARref), all other contours represent the delineations from the different radiation oncologists. **a** Brainstem, axial plane: the circumferential contour shows little variation; **b** cochlea, axial plane: two clinicians delineated the entire petrous part of the temporal bone; **c** cochlea wrongly delineated in axial plane; **d** glottic area, axial plane: difference in circumferential delineation, one clinician including the thyroid cartilage; **e** glottic area, sagittal plane: difference in cranial and caudal border; **f** mandible, axial plane: sometimes teeth are included; **g** oral cavity, sagittal plane: caudal border heterogeneity; **h** parotid gland, axial plane: inclusion of masseter muscle by one clinician and difference in medial border; **i** superior PCM, axial plane: anterior border heterogeneity; **j** middle PCM, sagittal plane: cranial border should be at the cranial edge of C3 but is delineated up to two vertebrae lower by some clinicians; **k** submandibular gland, axial plane: almost no variation in contours; **l** cranial edge of submandibular gland, axial plane: more variation is seen; **m** supraglottic larynx, sagittal plane: large variation in how it is contoured in both cranial and caudal borders; **n** supraglottic larynx, axial plane: air included around the epiglottic tip.

## Data Availability

The datasets used and/or analysed during the current study are available from the corresponding author on reasonable request.

## Abbreviations

DSC: Dice similarity coefficient
DVH: Dose volume histogram
HD95: 95^Th^ percentile Hausdorff distance
HNC: Head and neck cancer
ICG: International consensus guidelines
mm: Millimetre
MSD: Mean surface distance
IOV: Interobserver variability
OARref: Organs at risk reference delineation
OARs: Organs at risk
PCM: Pharyngeal constrictor muscle
RO: Radiation oncologist
RT: Radiotherapy

## References

[1] Bourhis J, Auperin A, Alfonsi M, Sunxu S, Rives M, Pointreau Y (2017). Dose escalation of radiotherapy (RT) for locally advanced head and neck carcinomas treated with concomitant chemotherapy (CT) and RT: Results of the GORTEC 2004–01 randomized trial. J Clin Oncol..

[2] Pignon J-P, le Maître A, Maillard E, Bourhis J (2009). Meta-analysis of chemotherapy in head and neck cancer (MACH-NC): An update on 93 randomised trials and 17,346 patients. Radiother Oncol.

[3] Nuyts S, Dirix P, Clement PMJ, Vander PV, Delaere P, Schoenaers J (2009). Impact of adding concomitant chemotherapy to hyperfractionated accelerated radiotherapy for advanced head-and-neck squamous cell carcinoma. Int J Radiat Oncol..

[4] Due AK, Vogelius IR, Aznar MC, Bentzen SM, Berthelsen AK, Korreman SS (2014). Recurrences after intensity modulated radiotherapy for head and neck squamous cell carcinoma more likely to originate from regions with high baseline [18F]-FDG uptake. Radiother Oncol.

[5] Bayman E, Prestwich RJD, Speight R, Aspin L, Garratt L, Wilson S (2014). Patterns of failure after intensity-modulated radiotherapy in head and neck squamous cell carcinoma using compartmental clinical target volume delineation. Clin Oncol [Internet].

[6] Grégoire V, Langendijk JA, Nuyts S (2015). Advances in radiotherapy for head and neck cancer. J Clin Oncol.

[7] Nutting CM, Morden JP, Harrington KJ, Urbano TG, Bhide SA, Clark C (2011). Parotid-sparing intensity modulated versus conventional radiotherapy in head and neck cancer (PARSPORT): a phase 3 multicentre randomised controlled trial. Lancet Oncol..

[8] Ghosh-Laskar S, Yathiraj PH, Dutta D, Rangarajan V, Purandare N, Gupta T (2015). Prospective randomized controlled trial to compare 3-dimensional conformal radiotherapy to intensity-modulated radiotherapy in head and neck squamous cell carcinoma: long-term results. Head Neck..

[9] Gupta T, Agarwal J, Jain S, Phurailatpam R, Kannan S, Ghosh-Laskar S (2012). Three-dimensional conformal radiotherapy (3D-CRT) versus intensity modulated radiation therapy (IMRT) in squamous cell carcinoma of the head and neck: a randomized controlled trial. Radiother Oncol.

[10] Rathod S, Gupta T, Ghosh-Laskar S, Murthy V, Budrukkar A, Agarwal J (2013). Quality-of-life (QOL) outcomes in patients with head and neck squamous cell carcinoma (HNSCC) treated with intensity-modulated radiation therapy (IMRT) compared to three-dimensional conformal radiotherapy (3D-CRT): evidence from a prospective randomized s. Oral Oncol.

[11] Mukesh M, Benson R, Jena R, Hoole A, Roques T, Scrase C (2012). Interobserver variation in clinical target volume and organs at risk segmentation in post-parotidectomy radiotherapy: can segmentation protocols help?. Br J Radiol.

[12] Cooper JS, Mukherji SK, Toledano AY, Beldon C, Schmalfuss IM, Amdur R (2007). An evaluation of the variability of tumor-shape definition derived by experienced observers from CT images of supraglottic carcinomas (ACRIN protocol 6658). Int J Radiat Oncol.

[13] Rasch C, Eisbruch A, Remeijer P, Bos L, Hoogeman M, van Herk M (2002). Irradiation of paranasal sinus tumors, a delineation and dose comparison study. Int J Radiat Oncol.

[14] Riegel AC, Berson AM, Destian S, Ng T, Tena LB, Mitnick RJ (2006). Variability of gross tumor volume delineation in head-and-neck cancer using CT and PET/CT fusion. Int J Radiat Oncol.

[15] Hermans R, Feron M, Bellon E, Dupont P, Van den Bogaert W, Baert AL (1998). Laryngeal tumor volume measurements determined with CT: a study on intra- and interobserver variability. Int J Radiat Oncol.

[16] van der Veen J, Gulyban A, Nuyts S (2019). Interobserver variability in delineation of target volumes in head and neck cancer. Radiother Oncol..

[17] Brouwer CL, Steenbakkers RJ, van den Heuvel E, Duppen JC, Navran A, Bijl HP (2012). 3D Variation in delineation of head and neck organs at risk. Radiat Oncol.

[18] Brouwer CL, Steenbakkers RJHM, Bourhis J, Budach W, Grau C, Grégoire V (2015). CT-based delineation of organs at risk in the head and neck region: DAHANCA, EORTC, GORTEC, HKNPCSG, NCIC CTG, NCRI, NRG oncology and TROG consensus guidelines. Radiother Oncol.

[19] Willems S, Crijns W, La Greca Saint-Esteven A, Van Der Veen J, Robben D, Depuydt T, et al. Clinical implementation of deepvoxnet for auto-delineation of organs at risk in head and neck cancer patients in radiotherapy. Vol. 11041 LNCS, Lecture Notes in Computer Science (including subseries Lecture Notes in Artificial Intelligence and Lecture Notes in Bioinformatics). 2018.

[20] van der Veen J, Willems S, Deschuymer S, Robben D, Crijns W, Maes F (2019). Benefits of deep learning for delineation of organs at risk in head and neck cancer. Radiother Oncol.

[21] Deeley MA, Chen A, Datteri R, Noble JH, Cmelak AJ, Donnelly EF (2011). Comparison of manual and automatic segmentation methods for brain structures in the presence of space-occupying lesions: a multi-expert study. Phys Med Biol.

[22] Christianen MEMC, Langendijk JA, Westerlaan HE, Water TA Van De, Bijl HP (2011). Delineation of organs at risk involved in swallowing for radiotherapy treatment planning. Radiother Oncol..

[23] Genovesi D, Perrotti F, Trignani M, Di Pilla A, Vinciguerra A, Augurio A (2015). Delineating brachial plexus, cochlea, pharyngeal constrictor muscles and optic chiasm in head and neck radiotherapy: a CT-based model atlas. Radiol Medica..

[24] Geets X, Daisne J-F, Arcangeli S, Coche E, De PM, Duprez T (2005). Inter-observer variability in the delineation of pharyngo-laryngeal tumor, parotid glands and cervical spinal cord: comparison between CT-scan and MRI. Radiother Oncol.

[25] Nelms BE, Tomé WA, Robinson G, Wheeler J (2012). Variations in the contouring of organs at risk: test case from a patient with oropharyngeal cancer. Int J Radiat Oncol..

