# Supplementary material for: Interobserver variability in organ at risk delineation in head and neck cancer
Source: Radiat Oncol. 2021 Jun 28;16:120. doi: 10.1186/s13014-020-01677-2 (PMC8240214; doi:10.1186/s13014-020-01677-2)
Supplement: Supplementary file 1 — Additional file 1. Survey and results from 14 radiation oncologists (RO). [file 13014_2020_1677_MOESM1_ESM.docx]

*Additional file 1*

*Survey questions*

# Do you use published guidelines to delineate organs at risk?

- Yes
- No
- If yes, please specify which one(s):

# Do you think there is a need for making and/or updating these delineation guidelines?

- Yes
- No
- If yes, please specify:_______________________________________________

*Answers from 14 radiation oncologists (RO)*

|  | Survey questions | Confirmed by no. of RO |
| --- | --- | --- |
| 1. | Do you use published guidelines to delineate organs at risk? | |
|  | - Yes | 13 |
|  | - - - - Brouwer et al. (18) | 5 |
|  | - - - - Brouwer et al. and Christianen et al. (21) | 1 |
|  | - - - - Genovesi et al. (22) | 1 |
|  | - - - - Not specified | 6 |
|  | - No | 1 |
| 2. | Do you think there is a need for making and/or updating guidelines? | |
|  | - Yes: 1 RO answered that guidelines for the brachial plexus needed clarification | 7 |
|  | - No | 7 |
