# Supplementary material for: Interobserver variability in organ at risk delineation in head and neck cancer
Source: Radiat Oncol. 2021 Jun 28;16:120. doi: 10.1186/s13014-020-01677-2 (PMC8240214; doi:10.1186/s13014-020-01677-2)
Supplement: Supplementary file 2 — Additional file 2. Patient characteristics. [file 13014_2020_1677_MOESM2_ESM.docx]

Additional file 2

Table 1 Patient characteristics

|  | **Patient 1** | **Patient 2** | **Patient 3** | **Patient 4** | **Patient 5** |
| --- | --- | --- | --- | --- | --- |
| **Site** | Oropharynx | Oropharynx | Supraglottis | Larynx | Hypopharynx |
| **TNM** | cT4bN0 | cT2N3b | cT2N0 | cT3N0 | cT2N2b |
| **Staging** | Stage IVb | Stage IVb | Stage II | Stage III | Stage IVa |
| **GTV volume primary** | 22.5cc | 20.1cc | 21.6cc | 2.3cc | 14.2cc |
| **GTV volume nodes** | - | 20.5cc | - | - | 8.1cc |

*TNM staging is given according to the last TNM staging system, TNM8*
