# Supplementary material for: Interobserver variability in organ at risk delineation in head and neck cancer
Source: Radiat Oncol. 2021 Jun 28;16:120. doi: 10.1186/s13014-020-01677-2 (PMC8240214; doi:10.1186/s13014-020-01677-2)
Supplement: Supplementary file 3 — Additional file 3. DSC and MSD of all OARS for all 5 patients. Every data point represents an organ at risk in one patient. [file 13014_2020_1677_MOESM3_ESM.docx]

Additional file 3

Figure 1 DSC and MSD of all OARS for all 5 patients. Every data point represents an organ at risk in one patient. Abbreviations: DSC: Dice similarity coefficient; mm: millimetre; MSD: mean surface distance; OARs: organs at risk
