# Supplementary material for: Interobserver variability in organ at risk delineation in head and neck cancer
Source: Radiat Oncol. 2021 Jun 28;16:120. doi: 10.1186/s13014-020-01677-2 (PMC8240214; doi:10.1186/s13014-020-01677-2)
Supplement: Supplementary file 4 — Additional file 4. Boxplots highlighting the differences between radiation oncologists using the guidelines from Brouwer et al., compared to radiation oncologists who use no or other guidelines. a Results of the dice similarity coefficient shows no significant difference between the two groups (p= 0.112). b Results for HD95 shows no significant difference between the two groups (p=0.219). [file 13014_2020_1677_MOESM4_ESM.docx]

Additional file 4

| a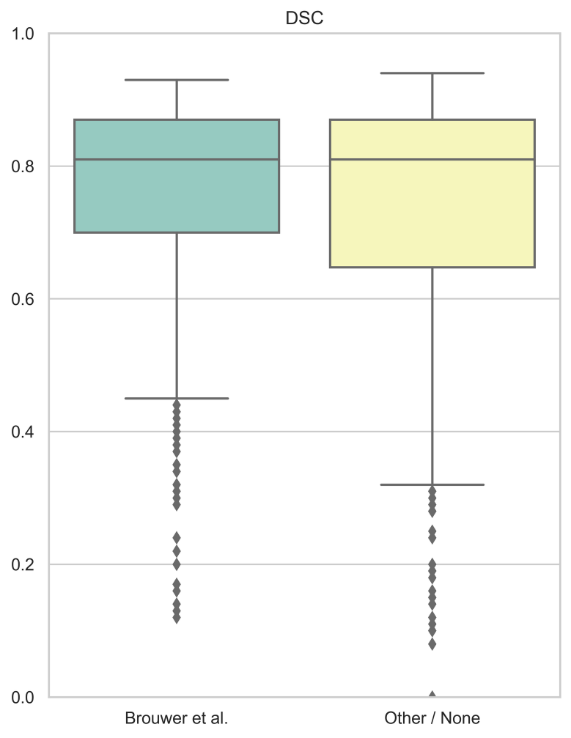 | b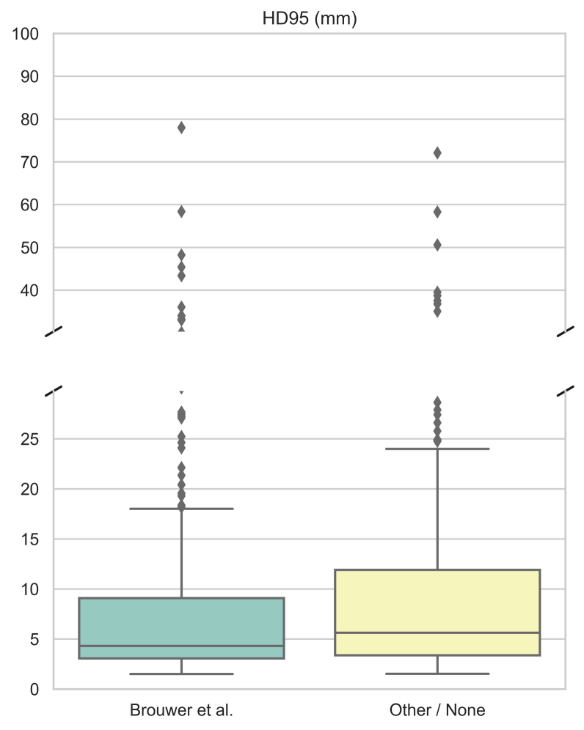 |
| --- | --- |

Figure 2 Boxplots highlighting the differences between radiation oncologists using the guidelines from Brouwer et al., compared to radiation oncologists who use no or other guidelines. (a) Results of the dice similarity coefficient shows no significant difference between the two groups (p= 0.112). (b) Results for HD95 shows no significant difference between the two groups (p=0.219). Abbreviations: DSC: Dice similarity coefficient; HD95: 95th percentile Hausdorff distance; mm: millimetre; RO: radiation oncologists.
