# Supplementary material for: Interobserver variability in organ at risk delineation in head and neck cancer
Source: Radiat Oncol. 2021 Jun 28;16:120. doi: 10.1186/s13014-020-01677-2 (PMC8240214; doi:10.1186/s13014-020-01677-2)
Supplement: Supplementary file 5 — Additional file 5. The boxplots depict the variation in volumes delineated by the different radiation oncologists for each patient separately. The boxplot shows the interquartile range (IQR), the median (horizontal line) and the minimum and maximum volume delineated (whiskers). OARref shows the organ at risk volume delineated according to the international consensus guidelines of Brouwer et al. [file 13014_2020_1677_MOESM5_ESM.docx]

Additional file 5

|  |  |  |
| --- | --- | --- |
|  |  |  |

Figure 3 The boxplots depict the variation in volumes delineated by the different radiation oncologists for each patient separately. The boxplot shows the interquartile range (IQR), the median (horizontal line) and the minimum and maximum volume delineated (whiskers). OARref shows the organ at risk volume delineated according to the international consensus guidelines of Brouwer et al. Abbreviations: IQR: interquartile range; OAR: organ at risk; PCM: pharyngeal constrictor muscle; SMG: submandibular gland.
