# Supplementary material for: Interobserver variability in organ at risk delineation in head and neck cancer
Source: Radiat Oncol. 2021 Jun 28;16:120. doi: 10.1186/s13014-020-01677-2 (PMC8240214; doi:10.1186/s13014-020-01677-2)
Supplement: Supplementary file 6 — Additional file 6. CT images showing interobserver variation for OAR contouring. The lilac volume is the reference delineation according to the guidelines (OARref), all other contours represent the delineations from the different radiation oncologists. a Brainstem, axial plane: the circumferential contour shows little variation; b cochlea, axial plane: two clinicians delineated the entire petrous part of the temporal bone; c cochlea wrongly delineated in axial plane; d glottic area, axial plane: difference in circumferential delineation, one clinician including the thyroid cartilage; e glottic area, sagittal plane: difference in cranial and caudal border; f mandible, axial plane: sometimes teeth are included; g oral cavity, sagittal plane: caudal border heterogeneity; h parotid gland, axial plane: inclusion of masseter muscle by one clinician and difference in medial border; i superior PCM, axial plane: anterior border heterogeneity; j middle PCM, sagittal plane: cranial border should be at the cranial edge of C3 but is delineated up to two vertebrae lower by some clinicians; k submandibular gland, axial plane: almost no variation in contours; l cranial edge of submandibular gland, axial plane: more variation is seen; m supraglottic larynx, sagittal plane: large variation in how it is contoured in both cranial and caudal borders; n supraglottic larynx, axial plane: air included around the epiglottic tip. [file 13014_2020_1677_MOESM6_ESM.docx]

Additional file 6

| a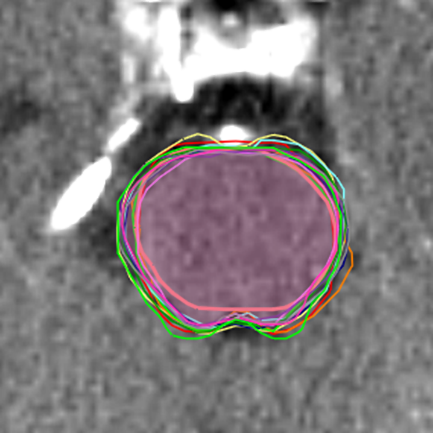 | b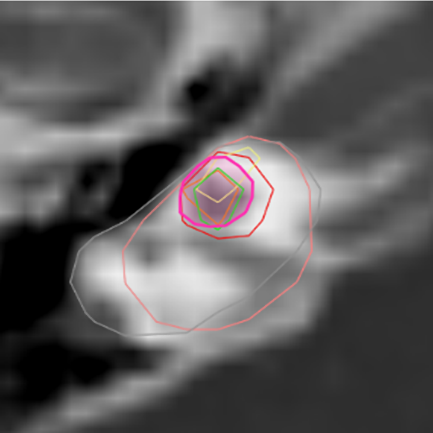 | c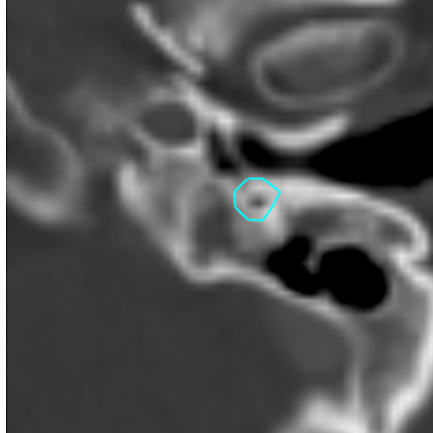 |
| --- | --- | --- |
| d 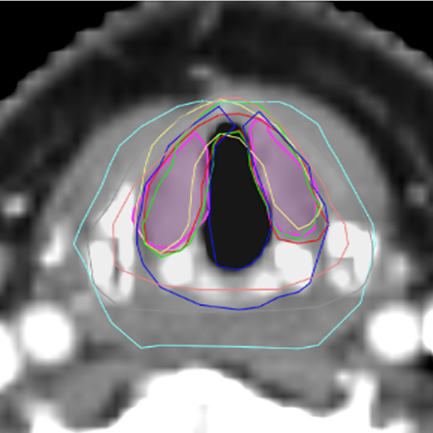 | e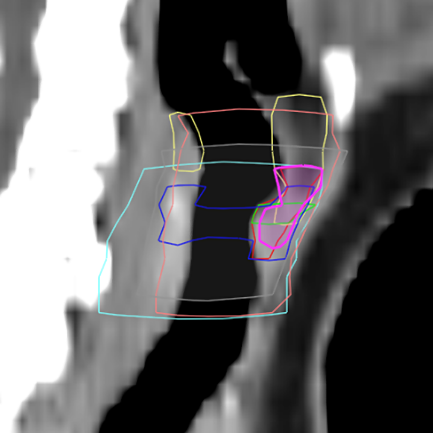 | f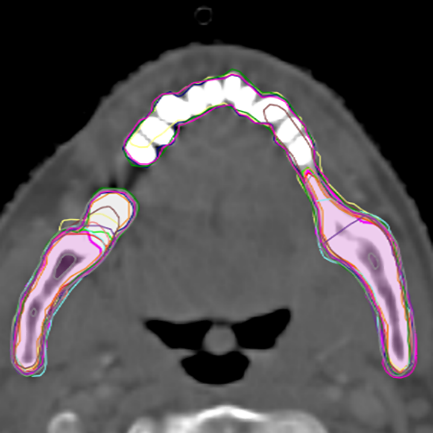 |
| g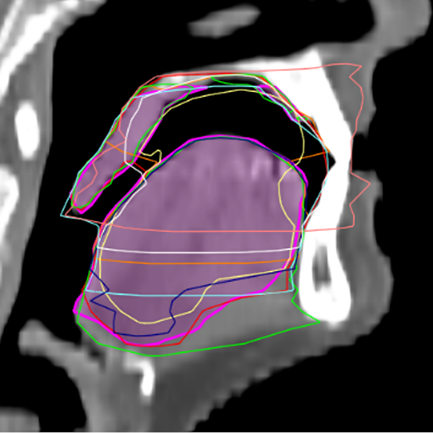 | h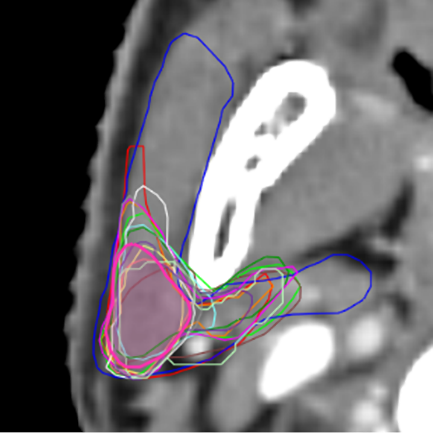 | i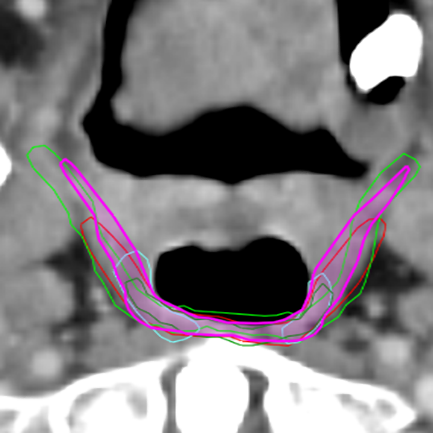 |
| j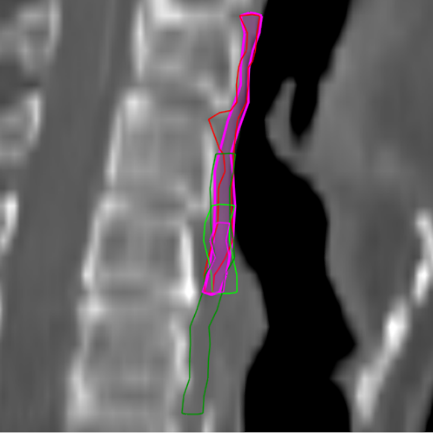 | k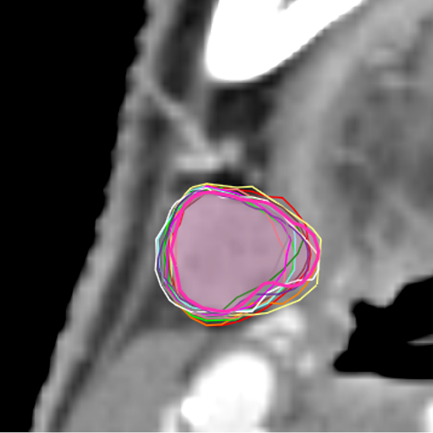 | l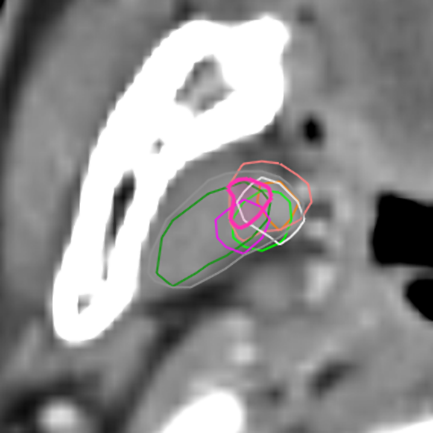 |
| m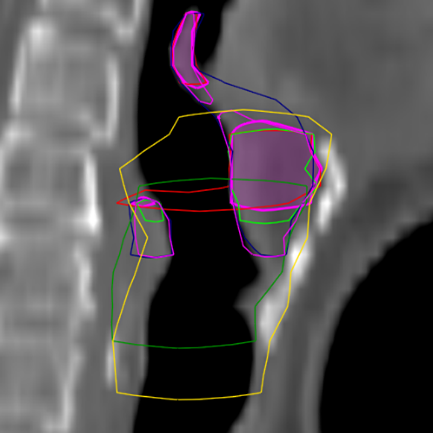 | n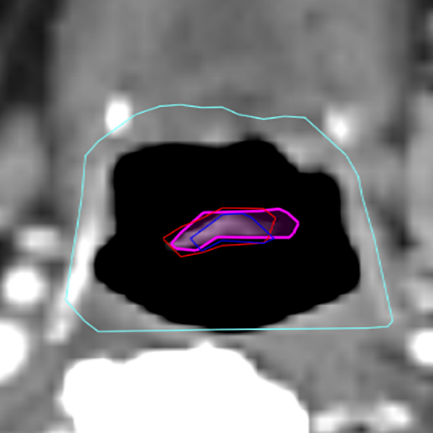 |  |

Figure 4 CT images showing interobserver variation for OAR contouring. The lilac volume is the reference delineation according to the guidelines (OARref), all other contours represent the delineations from the different radiation oncologists. (a) brainstem, axial plane: the circumferential contour shows little variation; (b) cochlea, axial plane: two clinicians delineated the entire petrous part of the temporal bone; (c) cochlea wrongly delineated in axial plane; (d) glottic area, axial plane: difference in circumferential delineation, one clinician including the thyroid cartilage; (e) glottic area, sagittal plane: difference in cranial and caudal border; (f) mandible, axial plane: sometimes teeth are included; (g) oral cavity, sagittal plane: caudal border heterogeneity; (h) parotid gland, axial plane: inclusion of masseter muscle by one clinician and difference in medial border; (i) superior PCM, axial plane: anterior border heterogeneity; (j) middle PCM, sagittal plane: cranial border should be at the cranial edge of C3 but is delineated up to two vertebrae lower by some clinicians; (k) submandibular gland, axial plane: almost no variation in contours; (l) cranial edge of submandibular gland, axial plane: more variation is seen; (m) supraglottic larynx, sagittal plane: large variation in how it is contoured in both cranial and caudal borders; (n) supraglottic larynx, axial plane: air included around the epiglottic tip. Abbreviation: OAR: organ at risk; OARref: reference contour; PCM: pharyngeal constrictor muscle.
